# Co-occurrence of suspected scoliosis and sagittal spinal deviations among early adolescents: a school-based cross-sectional study of prevalence and associated factors

**DOI:** 10.3389/fped.2026.1769144

**Published:** 2026-04-22

**Authors:** Patcharin Nilmart, Mantana Vongsirinavarat

**Affiliations:** 1 Faculty of Physical Therapy, Srinakharinwirot University, Ongkharak, Nakhon Nayok, Thailand; 2 Faculty of Physical Therapy, Mahidol University, Putthamonthon, Nakhon Pathom, Thailand

**Keywords:** adolescents, health behavior, musculoskeletal health, school health, scoliosis, spinal curvature

## Abstract

**Aims:**

This study aimed to investigate the prevalence and patterns of spinal postural abnormalities, particularly the co-occurrence of suspected scoliosis and sagittal spinal deviation, and to identify associated biological, behavioral, and psychosocial factors among early adolescents aged 12 to14 years.

**Methods:**

A school-based, cross-sectional study was conducted among 255 school children aged 12 to 14 years. The standardized postural assessments and validated questionnaires were used to obtain data on the spinal alignment, anthropometric measures, pain location, and behavioral and psychosocial characteristics. Frontal and sagittal deviations were assessed through standardized visual inspection procedures. Frontal deviations indicating suspected scoliosis were further examined using the Adam's forward bending test and the angle of trunk rotation (ATR) measured with a scoliometer. Behavioral variables included screen time and preferred posture during screen use, while psychosocial variables comprised knowledge, attitude, and practice (KAP) and self-postural awareness questionnaires. Associated factors were identified by performing comparative analyses and binary logistic regression.

**Results:**

Combined suspected scoliosis and sagittal spinal deviations were found in 21.2 percent of participants, with flattened thoracic curvature being the most frequent sagittal abnormality. Logistic regression revealed greater body height (AOR = 1.06, 95% CI: 1.02–1.11), lower KAP scores (AOR = 0.80, 95% CI: 0.72–0.89), while screen time (AOR = 1.22, 95% CI: 1.01–1.49) showed a more limited association with combined spinal deviations. Participants with suspected scoliosis reported more frequent upper back pain and lower self-postural awareness than those without deviations.

**Conclusion:**

A noteworthy proportion of early adolescents exhibited combined spinal deformities associated with biological and psychosocial–behavioral factors. Greater body height and lower KAP scores showed clearer associations with these conditions, whereas screen time demonstrated a tendency toward an association. These findings highlight the importance of incorporating posture assessment and health education into school-based initiatives. Improving early identification and encouraging healthy lifestyle behaviors may contribute to better spinal health and help reduce the risk of long-term musculoskeletal problems during adolescence.

## 1 Introduction

Early adolescence is considered a very important period of physical, behavioral, and musculoskeletal development (1). These changes can result in asynchronous development between the spine and surrounding tissues, which may be responsible for deviations in spinal curvature (2, 3).

Spinal deviations can occur in both the frontal and sagittal planes; however, much of the previous literature has targeted anomalies in one particular anatomical plane, such as scoliosis, forward head posture (FHP), or lumbar lordosis, which usually results in large variability in the reported prevalence rates (4–6). Such variability is influenced by assessment methods, type and location of spinal deviation, and the age group studied. Recent research has highlighted that spinal deviations frequently coexist. Dop et al. (2024) reported that 76% of school-aged children presented at least one postural defect and 70% exhibited two or more deformities, most commonly scoliosis, kyphosis, and lordosis, often related to sedentary lifestyle and poor sitting posture (7). Afanasieva et al. (2020) also found a high incidence of posture pathology among school-age children, up to 12%, showing idiopathic scoliosis, besides other types of sagittal misalignments (8). Adolescents with thoracic scoliosis tend to have reduced thoracic kyphosis and disturbed lumbosacral alignment, which indicates the kinetic interdependence between the segments of the spine (9). These findings underscore that spinal deviations are multidimensional rather than isolated phenomena. However, despite the increased recognition of the co-occurrence, detailed patterns and associated factors of combined spinal deviations are still inadequately described, particularly among early adolescents in developing countries. This puts into perspective the need for comprehensive screening approaches assessing both frontal and sagittal alignments in diverse contextual settings.

Both biological and behavioral factors have been associated with spinal misalignments (10). Among biological characteristics, sex and body mass index (BMI) have shown mixed associations, with some studies reporting a higher prevalence of scoliosis in female students (11, 12). In the realm of behavioral factors, physical activity has an inconclusive association with spinal deviations, given the inconsistent findings of previous studies (11, 13). Sedentary behaviors, especially screen time after school hours, have been significantly associated with an increased risk of developing spinal misalignment (14). According to the knowledge, attitude, and practice (KAP) model, behavioral outcomes are shaped by individual awareness and attitudes (15). Although the KAP framework has been related to postural health in older students (16), its psychosocial relevance to early adolescents remains underexplored.

Combined spinal deviations may lead to a higher risk of long-term musculoskeletal complications, including chronic back pain and reduced functional mobility (17). While individual spinal deformities have been the focus of considerable past research, the co-occurrence of frontal and sagittal plane abnormalities and their associated risk factors have been under-investigated. Both the prevalence and correlates of these deviations may be further contextualized by country-specific environments, ergonomic conditions, and lifestyle patterns, especially within the developing world where lifestyle changes are rapid. Therefore, the present study investigated the prevalence and patterns of spinal deviations among early adolescents and identified the biological, behavioral, and psychosocial factors associated with the co-occurrence of suspected scoliosis and sagittal deviations. In addressing this gap, the study aims to enhance understanding of multidimensional postural health among youth and to help inform targeted, school-based strategies for early identification and prevention within varied sociocultural contexts. It was hypothesized that suspected scoliosis and sagittal spinal deviations would co-occur to a measurable degree among early adolescents and that combined deviations of the spine would be associated significantly with biological, behavioral, and psychosocial factors.

## 2 Materials and methods

### 2.1 Study design and setting

This preliminary cross-sectional study was designed to examine the prevalence and associated factors of combined spinal deviations among early adolescents aged 12 to 14 years, recruited from four secondary schools located in the southern region of Thailand between July and October 2024. The study was conducted in accordance with the STROBE guidelines for cross-sectional studies. The study was part of a school-based screening program coordinated with the local educational authority. All procedures were approved by the institutional ethics review board, and permission to conduct the research was obtained from the participating schools.

### 2.2 Ethical considerations

The study protocol and consent procedures were reviewed and approved by the Human Research Ethics Committee at Walailak University, Thailand (Reference No. WUEC-23-103-02). All participants and their guardians provided written informed consent prior to participation.

### 2.3 Participants and sampling

To minimize potential selection bias, classrooms from grades 7 through 9 were randomly selected based on their availability during free periods. All the students in the selected classrooms received standardized announcements explaining the study objectives and procedures and were invited to voluntarily participate. Students who provided written informed consent and were available for physical assessment were included in the study. Exclusion criteria were limited to individuals with musculoskeletal conditions unrelated to postural abnormalities (e.g., joint disorders, bone fractures) or systemic diseases that could affect bone development (e.g., spinal infections, growth hormone deficiency, neuromuscular disorders), following established screening protocols.

The required sample size was calculated using the formula for an infinite population proportion. Based on a previously published survey study of scoliosis conducted in Thailand (18), the prevalence of spinal abnormalities was assumed to be approximately 21%. With a maximum tolerated error of 5%, the minimum required sample size was therefore estimated to be 255 participants. Moreover, an *a priori* power analysis for hypothesis testing was performed using G*Power software (version 3.1.9.7, Germany), based on a multiple linear regression model as an approximation for multiple logistic regression. Assuming a medium effect size (f^2^ = 0.15), a power of 0.9, an alpha error probability of 0.05, and eight predictors (representing key biological, behavioral, and psychosocial variables), the minimum required sample size was 136 participants. Therefore, to address both study objectives, estimating prevalence and identifying associated factors, a total of 255 participants was considered appropriate.

### 2.4 Study variables

Key study outcomes were obtained through standardized physical examinations and validated questionnaires. Demographic and anthropometric variables included age, sex, height, weight, and body mass index (BMI). Postural alignment was assessed through standardized physical examination. Symptom-related data included self-reported musculoskeletal pain and its location (neck, upper back, or lower back). Behavioral variables comprised daily screen time, preferred posture during screen use, and physical activity level. Psychosocial factors included knowledge, attitude, and practice (KAP) related to spinal posture and self-postural awareness.

#### 2.4.1 Postural assessment

Spinal alignment was assessed by a licensed physical therapist with 18 years of clinical experience in musculoskeletal practice who received standardized training prior to data collection. Assessments were conducted in line with the Society on Scoliosis Orthopaedic and Rehabilitation Treatment (SOSORT) consensus guidelines for school-based screening (19, 20). In agreement with these international recommendations, radiographic imaging was not conducted as this study was designed as a non-invasive, school-based screening for the early detection of postural deviations, rather than as a diagnostic confirmation process. The use of surface-based assessment methods, such as visual inspection, the Adam's Forward Bending Test, and scoliometer measurement of the angle of trunk rotation (ATR), has been widely accepted as an appropriate and ethical strategy in large-scale adolescent screening programs (19–22).

Lateral spinal deviation (suspected scoliosis) was examined in a standing position using a standardized screening protocol. Trunk asymmetry was first checked by visual inspection, followed by palpation of the spinous processes for the alignment of the thoracic and lumbar region. Subsequently, the Adam's Forward Bending Test was performed, and the ATR was measured with a scoliometer. Intra- and inter-rater reliability of ATR measurements using a scoliometer has been previously described as excellent and very good, respectively (23). Although both 5° and 7° cutoffs have been adopted in previous studies, ATR with ≥5° was used in this study, enabling the detection of early signs of spinal asymmetry with the highest sensitivity (24, 25). Participants with visible lateral deviation, a positive Adam's Forward Bending Test, and ATR ≥5° were classified as suspected scoliosis.

Sagittal alignment, including forward head posture (FHP), thoracic curvature abnormalities (hypo-/hyper-kyphosis), and lumbar curvature abnormalities (hypo-/hyper-lordosis), was assessed using a standardized visual screening protocol (26). Assessments were made in a static standing position with a posture grid serving as a visual reference. FHP was determined by anterior translation of the external auditory meatus relative to the acromion, with thoracic and lumbar curvature abnormalities determined qualitatively based on visual flattening or exaggeration of natural spinal curves relative to the grid's vertical and horizontal reference lines.

#### 2.4.2 Physical activity (PA)

The level of physical activity was quantified using the Thai version of the International Physical Activity Questionnaire-Short Form (IPAQ-SF), which is a self-report measure used to quantify the amount of PA engaged in over the previous seven days. Time spent engaging in vigorous activity, moderate activity, walking, and sitting was determined in minutes per day and frequencies per week from the questionnaire. The data were converted into metabolic equivalent task minutes per week (MET-min/week) and categorized into low, moderate, or high levels of PA according to standardized IPAQ criteria. The IPAQ-SF is widely used internationally and has been translated into multiple languages for use in diverse populations (27–29). The Thai version has demonstrated acceptable validity and reliability in previous studies, which may serve as an indicator of its suitability for measuring PA in similar contexts, particularly in large-scale observational studies (30).

#### 2.4.3 Knowledge, attitude, and practice (KAP) toward spinal posture

The KAP questionnaire was developed based on an extensive literature review and related theoretical frameworks (16, 31, 32). Content validity testing involved two rounds of evaluation by a panel composed of three experts in musculoskeletal physical therapy. In the first round, the experts evaluated the draft questionnaire for content relevance, wording clarity, and alignment with the study objectives. Feedback from this round was used to revise and refine item phrasing and response options. In the second round, the revised version was re-evaluated by the same panel to establish content validity using the Content Validity Index (CVI) approach. Items were rated as “relevant” or “not relevant,” and a minimum criterion of agreement by at least two out of three experts (CVI ≥ 0.67) was set for item acceptance. All revised items achieved full consensus, with 100% agreement among the experts in the second evaluation, confirming satisfactory content validity. Pilot testing was then conducted with a small group of adolescents to assess item clarity, feasibility, and completion time, leading to minor refinements before final implementation. The finalized instrument consisted of eight knowledge items, five attitude items, and seven practice items. Responses in the knowledge and attitude sections were rated on a three-point Likert scale (“agree,” “uncertain,” “disagree” = 2, 1, 0), while practice items were scored as “regularly,” “sometimes,” or “never” (2, 1, 0). Higher scores indicated greater knowledge, more positive attitudes, and healthier postural practices. A composite KAP score was computed by summing domain-specific scores across all three sections.

#### 2.4.4 Self-postural awareness

Self-awareness of spinal posture was assessed using a researcher-developed four-item questionnaire derived from standard postural screening criteria (19, 20, 26). Items addressed awareness of lateral deviation, forward head posture, thoracic curvature, and lumbar curvature. Each item was accompanied by a visual illustration of normal alignment to aid comparison. Participants rated their posture as “normal,” “abnormal,” or “unsure.” Correct self-assessments (compared to objective findings) were scored as one point; incorrect or unsure answers received zero points. A total score ≥3 indicated adequate self-postural awareness. Content validation of the questionnaire was conducted in two rounds by a panel of three experts in musculoskeletal physical therapy. In the first round, experts reviewed each item for relevance, wording clarity, and alignment with the study objectives. The questionnaire was revised according to their feedback. In the second round, the revised version was re-evaluated for content validity using an expert consensus approach. An agreement by at least two out of three experts (CVI ≥ 0.67) was set as the acceptance criterion. All revised items achieved 100% agreement among the experts in the second evaluation, confirming excellent content validity. The final questionnaire was subsequently pilot tested for clarity, interpretability, and feasibility before final implementation.

### 2.5 Data collection

All assessments and data collection were performed on school premises. Each participant attended an individual evaluation session lasting approximately 20 min. Data were collected in a standardized sequence to ensure consistency and minimize potential measurement bias.

**Figure 1:**
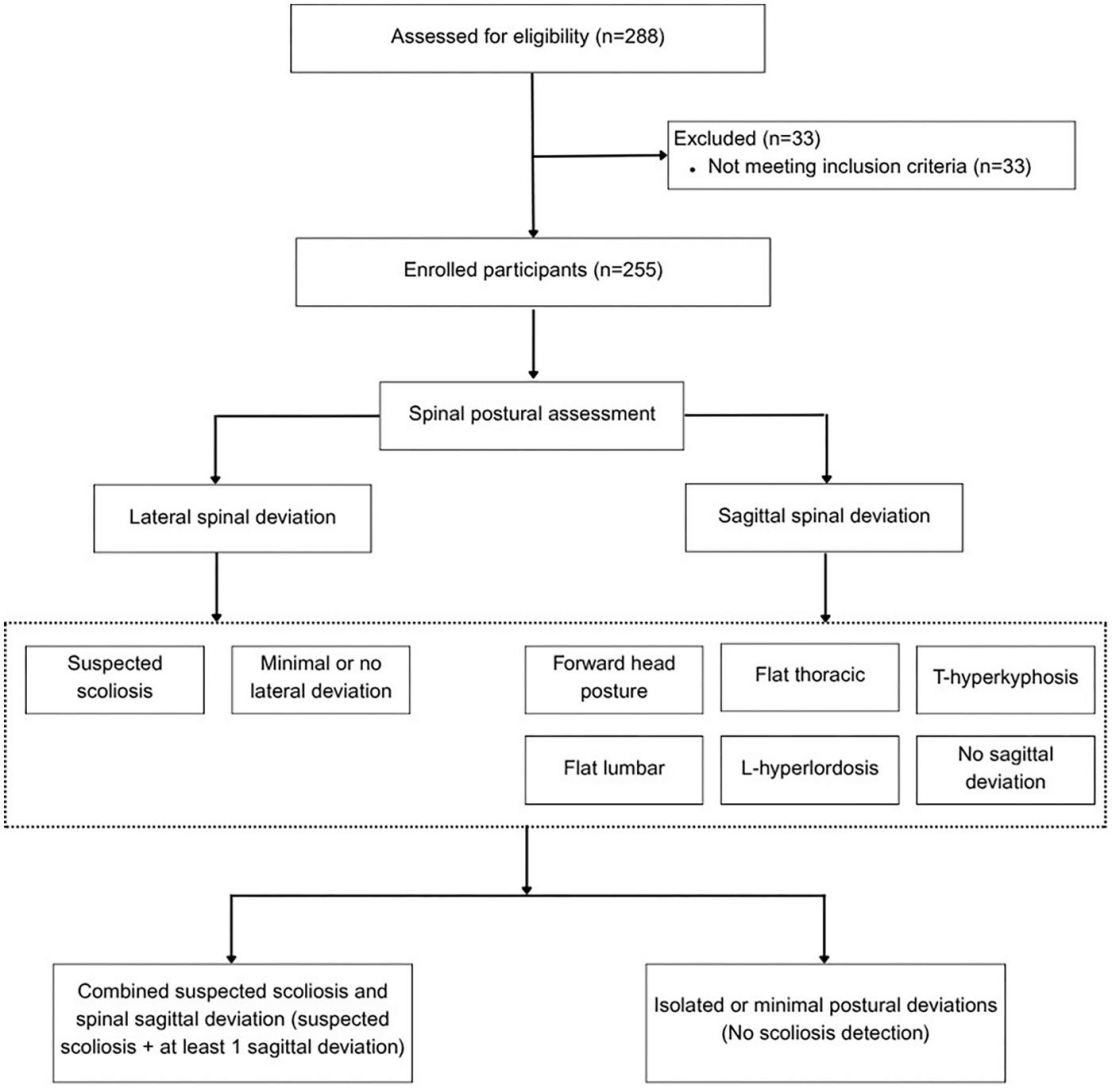
The group classification process.

First, anthropometric measurements (height, weight, and BMI) were obtained using calibrated equipment. Second, participants completed self-administered questionnaires assessing personal, pain location, and behavioral and psychosocial characteristics, including screen time, preferred posture during screen use, PA (IPAQ-SF), KAP, and self-postural awareness. Participants completed all questionnaires independently under the supervision of a trained research assistant, following the standardized administration procedure.In the final phase, postural assessments were performed in a private area to ensure comfort and confidentiality. The examiner was blinded to participants’ questionnaire responses to minimize observer bias. Spinal alignment was evaluated using visual inspection and instrumental assessment following SOSORT consensus guidelines for both the frontal and sagittal planes, as described in the study variable section. Participants were then categorized into two groups based on the severity of spinal deviation: (1) those with combined suspected scoliosis with postural sagittal deviations (defined as participants presenting with at least one sagittal plane deviation and clinical signs suggestive of scoliosis), and (2) those with isolated or minimal postural deviations (defined as participants without any signs of scoliosis). The group classification process is illustrated in Figure 1.

### 2.6 Statistical analysis

Statistical analyses were conducted using SPSS for Windows version 23 (IBM Corp., Armonk, NY, USA). A *p-*value of less than 0.05 was considered statistically significant. Descriptive statistics were used to summarize participant characteristics and the prevalence of spinal deviations. Frequencies and percentages were reported for categorical variables. The normality of continuous variables was assessed using the Kolmogorov–Smirnov test. For continuous variables, both means with standard deviations (SD) and medians with interquartile ranges (IQR) were presented due to non-normal distribution confirmed through normality testing. There were no missing data for any of the key variables included in the analysis. All quantitative variables including age, BMI, height, weight, KAP score, and screen time were treated as continuous variables.

Comparative analyses were performed to examine differences between participants with combined spinal deviation with scoliosis suspicion and those who demonstrated isolated or minimal deviations. The Mann–Whitney U test was used for continuous variables, as the variables were not normal distributed, while the Chi-square test was applied to categorical variables. For both tests, effect sizes were calculated to indicate the magnitude of group differences: r = Z/√N for the Mann–Whitney U test and Cramer's V for the Chi-square test. Variables showing a *p*-value < 0.25 in these preliminary analyses were entered into subsequent regression modeling.

Binary logistic regression analysis was conducted to identify factors associated with suspected scoliosis combined with sagittal spinal deviation. Univariate logistic regression was first performed to examine crude associations between each predictor and the dependent variable. Variables with *p* < 0.25 were subsequently entered into the multivariable logistic regression model, and adjusted odds ratios (AOR) with 95% confidence intervals (CIs) were calculated to quantify the strength and precision of associations (33). The multivariable model included sex, PA, body height, screen time, and KAP score as independent variables to generate AOR. Model fit was assessed using the Hosmer–Lemeshow goodness-of-fit test, and the explanatory power of the final model was evaluated using Nagelkerke's R^2^.

## 3 Results

**Figure 2:**
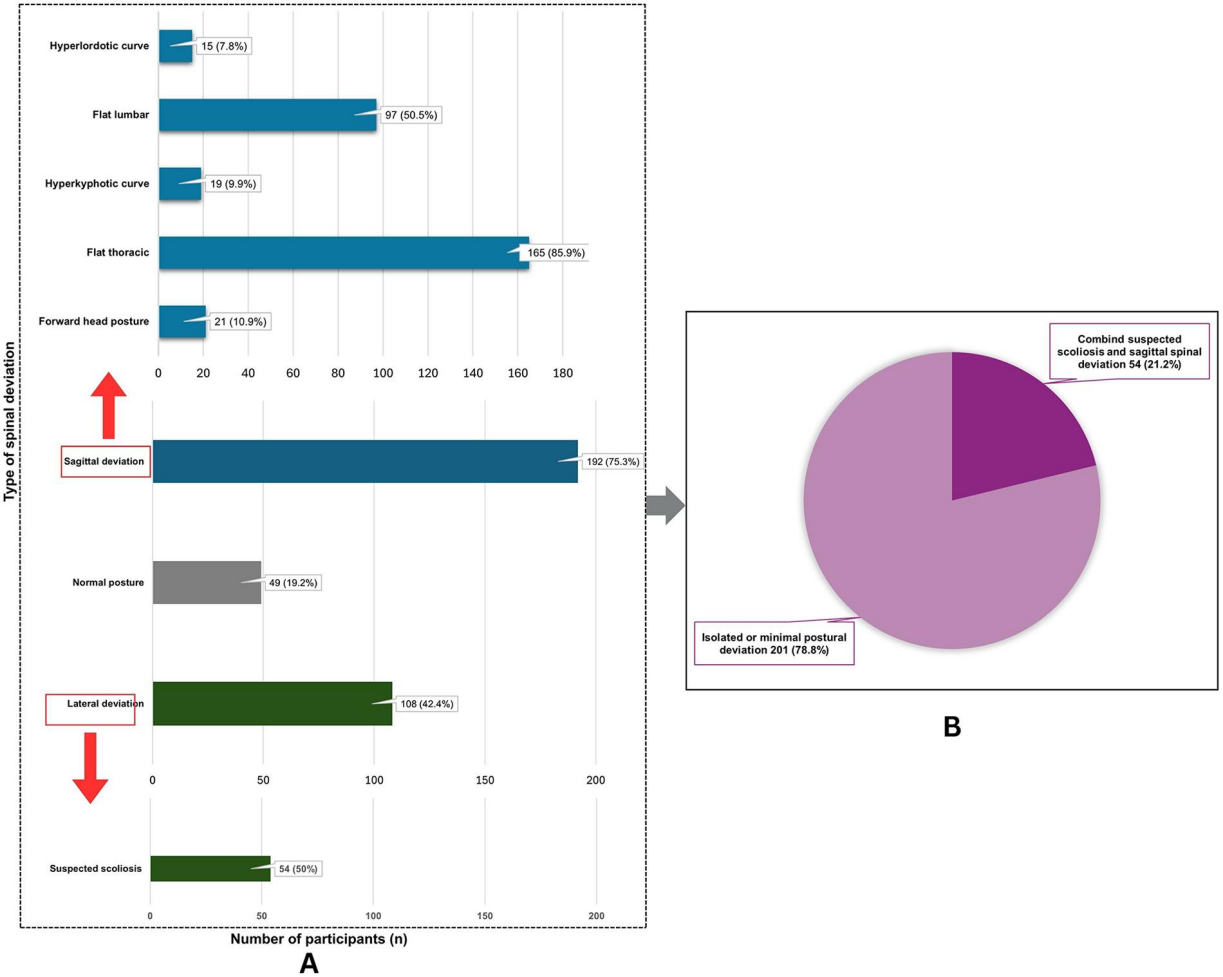
Distribution and co-occurrence of spinal postural deviations among early adolescents (*N* = 255). **(A)** Distribution of sagittal deviations and lateral deviations. Bars represent the number and percentage of participants with each deviation type. **(B)** Proportion of participants with combined suspected scoliosis and sagittal spinal deviation vs. those with isolated or minimal postural deviation.

**Figure 3:**
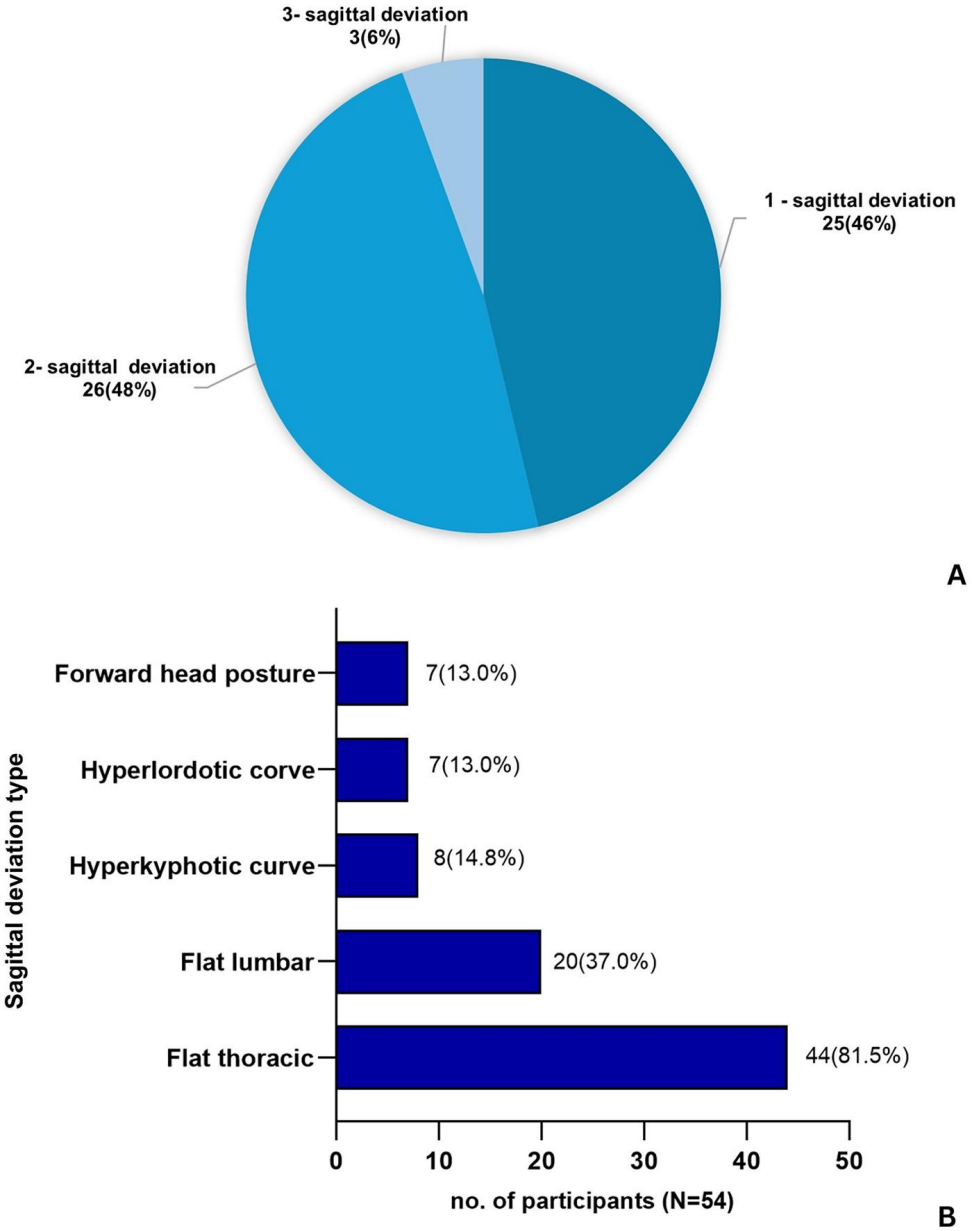
Patterns of sagittal spinal deviations among participants with suspected scoliosis (*n* = 54). **(A)** Number of sagittal deviation types identified in participants with combined suspected scoliosis (*n* = 54). **(B)** Frequency of specific sagittal deviation types among the same participants.

A total of 255 early adolescents (mean age = 13.5 years, 58% female) participated in this study. Most participants had normal body mass index (BMI) and low levels of PA (Table 1). Overall, 75.3% (*n* = 192) of participants presented with at least one sagittal spinal deviation, including forward head posture and thoracic or lumbar curvature abnormalities. Flat thoracic curvature was the most frequently observed sagittal abnormality. In addition, 42.4% (*n* = 108) exhibited lateral spinal deviation, with a mean angle of trunk rotation (ATR) of 4.5 ± 1.1 degrees among those affected. Of these, 54 participants (50.0%) demonstrated both a positive Adam's Forward Bending Test and an ATR of ≥5 degrees, indicating suspected scoliosis. A combination of suspected scoliosis and at least one sagittal deviation was found in 21.2% of the total sample (Figure 2). Among those with suspected scoliosis (*n* = 54), all had at least one coexisting sagittal deviation, most commonly a flat thoracic curvature (Figure 3).

**Table 1:** Demographic characteristics (*N* = 255).

Variables	Mean (SD)	Median (IQR)	Range	No (%)
Age (year)	13.5 (0.7)	14.0 (13.0–14.0)	12–14	
Weight (kg)	50.7 (13.0)	48.0 (42.0–57.0)	28–104	
Height (cm)	158.8 (7.9)	159.0 (153.0–165.0)	135–187	
BMI (kg/m^2^)	20.0 (4.6)	18.8 (16.7–21.6)	13.1–36.4	
Female				148 (58.0)
Physical activity level				
Low				143 (56.1)
Moderate				68 (26.7)
High				44 (17.3)

BMI, body mass index.

**Figure 4:**
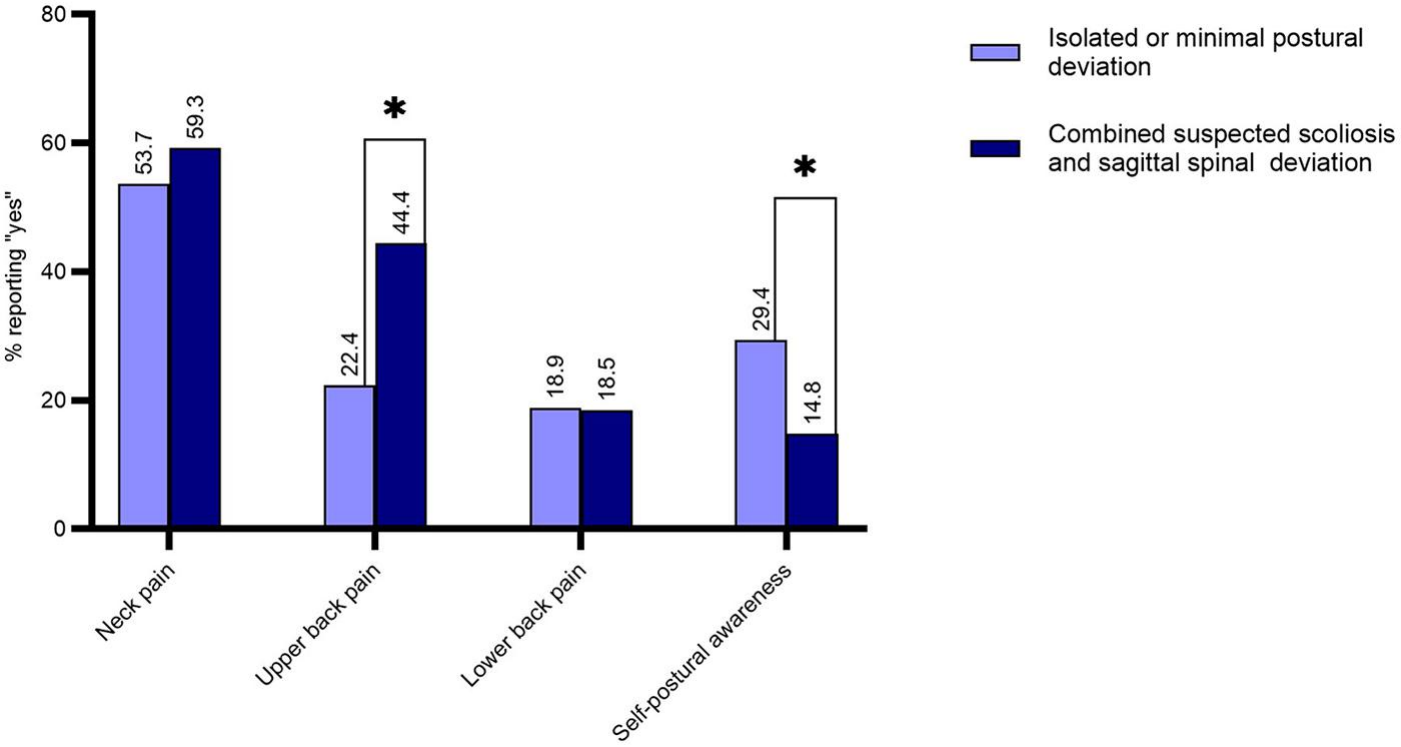
Comparison of pain distribution and self-postural awareness between groups.

Group comparisons showed significant differences in reported pain and self-postural awareness. Participants with combined suspected scoliosis and sagittal deviation reported upper back pain more frequently than those with isolated or minimal deviation (*p* = 0.002). No significant between-group differences were found for neck pain (*p* = 0.469) or lower back pain (*p* = 0.949). Self-postural awareness was significantly lower in the combined group (*p* = 0.031), although awareness was generally low in both groups, with fewer than one-third of participants recognizing their own postural alignment (Figure 4).

Comparisons of personal, behavioral, and psychosocial characteristics are presented in Tables 2, 3. Significant group differences were found for body height, KAP scores, and screen time (*p* < 0.05). Sex and physical activity level did not differ significantly (*p* > 0.05) but were retained for regression analysis based on the predefined inclusion threshold (*p* < 0.25). Effect sizes were also calculated and reported to indicate the magnitude of group differences. These variables were subsequently entered into the multivariable logistic regression model to explore their associations with combined spinal deviations.

**Table 2:** Comparison of continuous variables between groups (*N* = 255).

Variables	Isolated or minimal postural deviation (*n* = 201)		Combined suspected scoliosis and sagittal spinal deviation (*n* = 54)		*p*-value^a^	Effect size
	Mean (SD)^b^	Median (IQR)	Mean (SD)^b^	Median (IQR)		
Age (years)	13.5 (0.7)	14.0 (13.0–14.0)	13.4 (0.8)	14.0 (13.0–14.0)	0.785	0.02
Weight (kg)	50.7 (13.4)	48.0 (41.0–57.0)	50.6 (11.7)	47.5 (43–56.3)	0.926	0.01
Height (cm)	158.3 (7.8)	158.0 (153.0–164.5)	160.9 (8.0)	160.5 (155.8–165.0)	0.032*	0.13
BMI (kg/m^2^)	20.1 (4.7)	18.9 (16.8–22.0)	19.5 (4.2)	18.6 (16.4–20.8)	0.379	0.06
KAP (score)	28.7 (3.5)	29.0 (26.0–31.0)	26.6 (3.0)	27.0 (25.0–29.0)	<0.001*	0.27
Screen time (hour/ day)	4.88 (1.8)	5.0 (4.0–6.0)	5.7 (1.2)	5.0 (5.0–6.0)	0.001*	0.21

BMI, body mass index; KAP, knowledge, attitude, and practice.

a Comparisons between groups were performed using the Mann–Whitney U test based on median (IQR) values.

b Mean (SD) values are also presented for descriptive purpose.

*Significant difference at *p*-value < 0.05.

**Table 3:** Comparison of categorical variables between groups (*N* = 255).

Variables	Subgroup	Total (*N*)	Isolated or minimal postural deviation (*n* = 201) n(%)	Combined suspected scoliosis and sagittal spinal deviation (*n* = 54)	*χ*^2^ (df)	*p*-value^a^	Effect^b^ Size
Sex	Male	107	79 (73.8)	28 (26.2)	2.75 (1)	0.097	0.10
	Female	148	122 (82.4)	26 (17.6)			
PA level	Low	143	118 (82.5)	25 (17.5)	5.24 (2)	0.073	0.14
	Moderate	68	47 (69.1)	21 (30.9)			
	High	44	36 (81.8)	8 (18.2)			
Preferred Posture during screen time	Neutral sitting posture	44	34 (77.3)	10 (22.7)	2.09 (4)	0.718	0.09
	Neck flexed sitting posture	102	79 (77.5)	23 (22.5)			
	Supine position	57	46 (80.7)	11 (19.3)			
	Side-lying position	14	13 (92.9)	1 (7.1)			
	Prone position	38	29 (76.3)	9 (23.7)			

PA, physical activity.

a Group differences analyzed using the Chi-square test.

b Cramer's V.

Binary logistic regression analysis identified three independent predictors of combined suspected scoliosis and sagittal spinal deviation: greater body height (AOR = 1.06, 95% CI: 1.02–1.11, *p* = 0.006), lower KAP scores (AOR = 0.80, 95% CI: 0.72–0.89, *p* < 0.001), and longer screen time (AOR = 1.22, 95% CI: 1.01–1.49, *p* = 0.043). For screen time, the confidence interval was close to the null value, indicating a weak association. The final model explained 20.8% of the variance (Nagelkerke R^2^ = 0.208) and demonstrated acceptable model fit (*p* = 0.823), indicating a moderate level of explanatory power (Table 4).

**Table 4:** Logistic regression analysis identifying factors associated with combined suspected scoliosis and sagittal spinal deviation.

Variable	Univariate Logistic Regression		Multiple Logistic Regression	
	OR (95% CI)	*p*-value	AOR (95% CI)	*p*-value
Sex	1.66 (0.91–3.04)	0.099	-	-
Height (cm)	1.04 (1.00–1.08)	0.037*	1.06 (1.02–1.11)	0.006*
KAP (score)	0.84 (0.76–0.92)	<0.001*	0.80 (0.72–0.89)	<0.001*
Screen Time (hour/ day)	1.30 (1.09–1.56)	0.004*	1.22 (1.01–1.49)	0.043*
PA level (Moderate vs. Low)	0.95 (0.40–2.30)	0.915	0.733 (0.28–1.91)	0.526
PA level (High vs. Low)	2.01 (0.80–5.06)	0.138	1.91 (0.70–5.20)	0.206

OR, odds ratio; AOR, adjusted odds ratio; CI, confidence interval; KAP, knowledge-attitude-practice; PA, physical activity.

*Significant difference at *p*-value < 0.05.

## 4 Discussion

The findings of this study demonstrated that 21.2% of participants presented with combined suspected scoliosis and sagittal spinal deviation. This result indicates a significant burden of postural abnormalities in early adolescents and underscore the need for multiple spinal deviation assessment simultaneously, which has rarely been considered so far in scoliosis prevalence studies.

Previous studies have reported a wide range of scoliosis prevalence rates, which may be partly related to differing screening tools, diagnostic thresholds, and sample characteristics. A previous study of Norwegian children aged 12 years found that the prevalence of scoliosis with a primary major curve greater than 10 degrees on radiographs was 0.55%, while curves greater than 20 degrees were observed in only 0.13% of cases (34). In contrast, a recent study using postural observation with an angle of trunk rotation (ATR) greater than 5 degrees reported prevalence rates ranging from 2.56% to 8.75%, depending on age and sex (35). The Thai experience also includes a previous school-based survey of 10- to 15-year-old students, which similarly reported (18), and therefore further establishes local consistency with the results of the current study. Although a standing radiograph with Cobb angle measurement remains the diagnostic gold standard (36), radiographic confirmation was not feasible in this school-based screening. Moreover, the SOSORT has recommended minimizing radiation exposure during pediatric screening (37). Therefore, visual inspection by an experienced physiotherapist, enhanced by scoliometer measurement, was used to determine the presence of postural deviations and guide recommendations for early conservative management. An ATR of 5 degrees was also considered clinically meaningful and has been suggested as a practical threshold for identifying candidates for early conservative care (38). Based on this criterion, approximately 20% of participants with suspected scoliosis in this study would warrant further evaluation.

Sagittal thoracic deviations were the most frequent postural misalignment, particularly flattened thoracic curvature or thoracic hypokyphosis. Indeed, all participants with suspected scoliosis exhibited deviations in both the frontal and sagittal planes, suggesting that global spinal alignment was altered. However, flat thoracic curvature was also seen among participants without suspected scoliosis, consistent with previous reports showing that sagittal deviations such as hypokyphosis can occur in both scoliotic and non-scoliotic adolescents. Thoracic hypokyphosis may not directly cause scoliosis but could serve as a compensatory mechanism that facilitates axial spinal rotation (39). Importantly, sagittal deviations including hypokyphosis have been associated with musculoskeletal complaints, particularly upper back pain (40). In the present study, participants with combined sagittal spinal deviation and suspected scoliosis reported a higher prevalence of upper back pain compared to those without scoliosis. This may reflect a multifactorial biomechanical stress from frontal and sagittal misalignment, muscular imbalance, and postural strain (41–43).

In multivariable analysis, biological, behavioral, and psychosocial attributes emerged as the key predictors of combined suspected scoliosis and sagittal spinal deviation. Greater body height was significantly associated with higher odds of the condition. These results are in accordance with previous data indicating that taller adolescents may be more susceptible to a disturbance in the spinal alignment during rapid growth spurts, especially if neuromuscular control is too weak to stabilize the new biomechanical alignment pattern of the spine (44). Furthermore, a previous study reported a positive association between body height and the severity of spinal deformities (45). In the current study, however, no other biological attributes were significantly associated with suspected scoliosis and sagittal spinal deviation, including sex and BMI. This may be partly explained by the homogeneity of the sample, as participants had generally normal BMI values. Previous research has reported that a low BMI may be associated with the presence of scoliosis, although the underlying mechanism remains unclear (12). Another study found that BMI did not independently predict sagittal spinal alignment among children and adolescents after adjusting for age, implying that growth-related changes account for much of the variability (46). Although sex was not a significant predictor in this study, previous studies have consistently demonstrated higher scoliosis prevalence and greater curve magnitude among females (24, 47, 48). Sex hormones have been proposed to play a role in scoliosis development, potentially explaining the higher prevalence among girls (49). Moreover, gender-related differences in sagittal curvature have been reported, with males more likely to exhibit thoracic kyphosis and females showing greater lumbar lordosis (50). The lack of sex-related differences in this study may be due to the narrow age range (12–14 years), which limited variability in developmental stages. Sex-related effects that typically appear across broader age spans may therefore not have been detected, as reported in studies involving wider age groups.

In addition, lower KAP scores indicated a higher risk of spinal deviation, suggesting the relevance of posture-related education at this age. This is also supported by previous findings that people who are poorly informed about health issues are less likely to use protective behavior and thus have a heightened risk for musculoskeletal disorders (51, 52). The association between KAP scores and abnormal spinal alignment supports the inclusion of posture education in school curricula as a means of maintaining spinal health and reducing the burden of musculoskeletal disorders (53). Lastly, higher screen time was identified as a behavioral predictor for combined spinal deviations; however, the confidence interval close to the null value suggests that this association should be interpreted with caution. Although this finding indicates a weak association, a previous study has suggested that prolonged sedentary behavior, sustained trunk flexion, and postural asymmetry during screen use may contribute to spinal misalignment (54). Furthermore, Zhu et al. (2023) reported that excessive screen time was a risk factor for adolescent idiopathic scoliosis (55). The potential effects of screen time on combined spinal abnormalities may require long-term follow-up.

Self-postural awareness is defined as the ability to sense the posture of one's own body in real time. In the present study, self-postural awareness was significantly lower for participants with combined suspected scoliosis and sagittal spinal deviation compared with those with only minimal or no postural abnormalities. This finding is consistent with previous research indicating that adolescents with idiopathic scoliosis often fail to accurately perceive their spinal curvature (56). Body awareness has also been found to be associated with musculoskeletal pain and the emotional status of a person (57, 58). A lack of awareness might delay the initiation of early intervention and lead to persisting postural malalignment or chronic symptoms. Furthermore, self-concept and body perception have close links with emotional regulation and psychological resilience during adolescence. As health behaviors established during this life period often continue into adulthood (59), educational approaches that improve awareness of normal and abnormal posture may be useful. Inadequate knowledge about spinal alignment can reduce adolescents’ perception of postural abnormalities, while school-based interventions have been found to potentially improve posture-related behavior (60).

These findings may have implications for the early identification and targeting of interventions in the prevention of spinal abnormalities among early adolescents. The high prevalence of spinal deviations, including the co-occurrence of scoliosis and sagittal misalignment, may indicate the relevance of a comprehensive postural examination. School-based screening by a trained professional, like a physiotherapist, could be one feasible and contextually appropriate strategy for the early detection of postural abnormalities. Behavioral and psychosocial factors, including poor KAP, screen-related behaviors, and low self-postural awareness, may further support the inclusion of posture education and behavior-oriented interventions within school-based programs. These strategies may help prevent the progression of spinal deformities and could reduce the burden of related musculoskeletal symptoms. Prevention-based approaches are particularly useful in developing countries due to the restricted early clinical care caused by limited financial and human resources. Low-cost education-based initiatives could help to enhance spinal health and ensure long-term musculoskeletal well-being among adolescents.

There were some limitations. First, the cross-sectional design of this study limits the establishment of any causal relationship between behavioral or perceptual factors and spinal abnormalities. Second, the sample was drawn from schools within a single province and may affect the generalizability of findings to broader adolescent populations in Thailand or other cultural contexts. Third, self-reported measures of physical activity, screen time, and KAP are potentially vulnerable to recall or social desirability biases. Lastly, spinal alignment assessment in this study relied primarily on visual inspection procedures. Although standardized protocols and experienced physiotherapists were employed to minimize observer bias, this method is inherently subjective and may lack the precision of instrument-based evaluations. Future studies should adopt objective measurement tools such as 3D posture analysis systems, or radiographic validation to enhance accuracy and reproducibility. Moreover, longitudinal studies are recommended to explore how biological growth factors, behavioral habits such as screen use, and psychosocial attributes like postural awareness interact to influence the progression or potential reversibility of spinal deviations during adolescence. In addition, school-based posture education programs should be further explored to confirm and address postural awareness problems among students.

In conclusion, this study revealed a considerable prevalence of suspected scoliosis and postural deviations among early adolescents. Biological, behavioral, and psychosocial variables, including body height, screen time, and KAP score, were associated with suspected scoliosis and postural deviation conditions. It further revealed significant inequalities between participants with and without suspected scoliosis regarding the presence of upper back pain and self-postural awareness levels. Such findings may be useful in the elaboration of school-based screening programs and health education to provide incentives for postural and healthy lifestyle awareness. Enhancing early identification and preventive measures could help in decreasing the burden of musculoskeletal disorders throughout adolescence.

## Data Availability

The raw data supporting the conclusions of this article will be made available by the authors, without undue reservation.
